# Satisfaction and quality of life with palatal positioned implants in severely atrophic maxillae versus conventional implants supporting fixed full-arch prostheses

**DOI:** 10.4317/medoral.20706

**Published:** 2015-06-27

**Authors:** Eugenia Candel-Marti, David Peñarrocha-Oltra, Maria Peñarrocha-Diago, Miguel Peñarrocha-Diago

**Affiliations:** 1 Professor of the Master in Oral Surgery and Implantology, Stomatology Department, Faculty of Medicine and Dentistry, University of Valencia, Valencia, Spain; 2 Full Professor of Oral Surgery, Stomatology Department, Faculty of Medicine and Dentistry, University of Valencia, Valencia, Spain; 3 Chairman of Oral Surgery, Director of the Master in Oral Surgery and Implantology, Stomatology Department, Faculty of Medicine and Dentistry, University of Valencia, Valencia, Spain

## Abstract

**Background:**

To evaluate satisfaction and quality of life in patients with palatal positioned implants supporting fixed full-arch prostheses to rehabilitate edentulous maxillae with horizontal atrophy and compare them with conventional well-centered implants placed in non-atrophic supporting fixed full-arch prostheses.

**Material and Methods:**

A clinical retrospective study was performed of patients that were rehabilitated with full-arch fixed implant-supported maxillary prostheses and had a minimum follow-up of 5 years after implant loading. Patients were divided into 2 groups: patients with class IV maxilla according to Cawood and Howell and treated with palatal positioned implants (test) and with class III maxilla and treated with implants well-centered in the alveolar ridge and completely surrounded by bone (control). Ten-cm visual analogue scales (VAS) (range 1-10) and the OHIP-14 (Oral Health Impact Profile) questionnaire were used respectively to estimate patient satisfaction and quality of life after implant therapy. Statistical analysis was performed applying Mann-Whitney Test using alpha set at 0.05.

**Results:**

Mean global and specific satisfaction – except for self-esteem – were superior for the test group than the control group, although differences were not statistically significant. Regarding quality of life, the reported incidence of problems was lower in the test group for all the studied ítems except for ‘problems at work’. However, differences were not statistically significant in any case.

**Conclusions:**

Despite the limitations of the study (retrospective and nonrandomized design) the results suggest that the prosthesis design needed to rehabilitate palatally positioned implants (more coverage of palate) does not lead to lower satisfaction and quality of life of patients, compared to patients treated with implants placed centered and conventional design prostheses that do not cover the palate.

**Key words:**Atrophic maxilla, palatal implants, satisfaction, quality of life.

## Introduction

According to the original protocol by Brånemark, dental implants should be placed upright, centered in the bone crest and completely surrounded by bone (1). This position can only be achieved in class III maxillae according to Cawood and Howell (2); i.e., maxillae with enough bone height and width. In class IV maxillae, where there is sufficient bone height but insufficient bone width, placement of dental implants completely surrounded by bone is complicated. To solve or bypass this situation, numerous surgical techniques have been proposed (3-6). These methods can be classified into bone grafting techniques (i.e., guided bone regeneration or block grafts (4) and modifications of the original implant insertion protocol that avoid bone grafting by using areas of residual bone (i.e., zygomatic implants (5), pterygoid implants (6), implant insertion in the maxillary tuberosity (7), tilted and palatal implants) (8-10).

An alternative in maxillae with a narrow residual crest (width < 4 mm) is the insertion of implants in a palatal position. In this cases Branemark *et al*. (7) suggested the anchorage of implants in the residual palatal bone. This modification of the insertion technique allows to have 2 mm of buccal bone even in atrophic ridges, while 2 to 5 implant threads are left exposed and covered with particulate bone graft (8). Peñarrocha *et al*. (8) studied 69 patients treated with 330 implants placed in palatal position and after 2 years of follow-up reported a success rate of 97.8%. In other study of Peñarrocha *et al*. (9), they evaluate the 5-year outcome of a previously reported case series (8) of patients with severely atrophic maxillae treated with palatally positioned implants and fixed full-arch rehabilitations, and reported a success rate of 98,7 %.

Rebuilding the atrophic maxilla with bone graft permits placing the implants in an ideal position from a prosthetic point of view. On the contrary, palatal positioned implants have to be rehabilitated with a prosthetic structure that has a palatal emergence. This could be associated with problems with mastication or phonetics or with discomfort for the patient. A previous report (8) addressed satisfaction with this kind of therapy, but no control group was involved.

The aim of the present study was to compare patient satisfaction and quality of life with full-arch fixed prostheses supported by palatal positioned implants or by conventional implants placed well-centered in the alveolar crest after a minimum follow-up of 5 years after prosthetic loading.

## Material and Methods

- Study design

A clinical, controlled, retrospective study was performed in the Oral Surgery and Implant Dentistry Division of the University of Valencia between January and December 2013. The research was performed following the principles of the Declaration of Helsinki on research involving human beings. Accordingly, all patients were informed about the study and they were asked to sign an informed consent document before being included. The study design was approved by the ethical review board of the University of Valencia (Ref: H1330446292077).

A chart-review was performed to retrospectively select patients according to the following criteria.

 * Inclusion criteria:

- Rehabilitation of the edentulous maxillae with fixed implant-supported prosthesis

- No previous bone grafting procedure to reconstruct atrophic alveolar ridges for implant insertion

- Minimum follow-up of 5 years after implant loading

* Exclusion criteria:

- Failure to attend scheduled control visits

- Refereed patients not being controlled at the Oral Surgery and Implant Dentistry Division

- Patients not agreeing to participate in the study

 * Included patients were divided into 2 study groups.

- Test group: patients with class IV maxilla according to Cawood and Howell (2) and treated with palatal positioned implants in the anterior and premolar regions.

- Control group: patients with class III maxilla according to Cawood and Howell (2) and treated with implants well-centered in the alveolar ridge and completely surrounded by bone.

- Treatment procedures

A clinical and radiographic examination was performed of all the patients, including panoramic radiography and computed tomography for surgical planning. All surgeries were performed by the same surgeon under local anesthesia with articaine 4% with epinephrine 1:100,000 (Inibsa, Lliça of Vall, Barcelona, Spain) and / or sedation with propofol solution of 1%; blood pressure, pulse and oxygen monitoring was performed by an anesthesiologist. All implants were Phibo® TSA with Avantblast Surface (Phibo Dental Solutions, Senmenat, Barcelona, Spain).

Test group: Surgical procedures for the rehabilitation of atrophic maxillae with palatal positioned implants were detailed in a previous report (8). Implants in the anterior and premolar regions were placed in palatal position, with 2 to 5 threads exposed on the palatal side. Exposed thread were covered with autologous particulate bone (when available) and Bio-Oss (Geistlich, Wolhusen, Switzerland).

Control group: Implants were placed well-centered in the alveolar ridge and completely surrounded by bone.

Sutures were removed 1 week after the surgery. Prosthesis fabrication began 3 months after implant placement. Fixed metal-ceramic prostheses were placed when the interoclusal space, the inter maxillary relation and the patient’s lip support were adequate. Screwed metal-resin prostheses were used when the interocclusal space was excessive or to compensate for lack of lip support. All patients were included in a maintenance program with controls visits involving professional prophylaxis every 6 months.

- Data collection

Ten-cm visual analogue scales (VAS) (range 1-10) and the OHIP-14 (Oral Health Impact Profile) (11) questionnaire were used respectively to estimate patient satisfaction and quality of life after implant therapy.

General satisfaction with the implant-retained prosthesis and specific satisfaction regarding comfort, mastication, phonetics, aesthetics, ease of cleaning and self-esteem were assessed using 10-cm visual analogue scales (VAS). The anchor words were “totally dissatisfied” and “completely satisfied.” Subjects were asked to draw a vertical line at a point on the horizontal line which best represented their response (8).

The Spanish validated version of the OHIP -14 questionnaire was used to assess patient quality of life (11). The full OHIP consists of 49 items that cover seven domains: functional limitation, physical pain, psychological discomfort, physical disability, psychological disability, social disability, and handicap. Locker and Allen (12) derived a subset of 14 of the original 49 items that can be used in any situation where a shorter version is deemed adequate. Responses to this scale are based on a Likert format, with a five-point ordinal scale ranging from “never” (coded 1) to “very often” (code 5) (13).

- Statistical analysis

Statistical analysis was performed using non-parametric tests due to the patient sample size. The Chi2 test and the Mann-Whitney test (MW) were used to evaluate homogeneity within the two groups in terms of a series of demographic and clinical parameters. The MW test for independent samples was used to assess differences in patient satisfaction and quality of life between the two groups. Statistical analysis was completed using SPSS 17.0 software (SPSS Inc., Chicago, IL) with alpha set to 0.05. A biostatistician with expertise in dentistry analyzed the data without knowledge of group assignment.

## Results

The chart review yielded 66 patients with 457 implants fulfilling the inclusion criteria. Nine patients were excluded: 4 due to failure to attend scheduled control visits and 5 because they were refereed patients not being controlled at the University. A total of 57 patients - 32 belonging to test group and 25 to the control group - were finally included. The mean follow-up period was 6.5 ± 1.3 years (range 5-11).

Patients from the test group received 225 implants. 161 were palatal positioned and were thus included, and 64 were excluded: 47 were placed well-centered in molar regions, 9 were pterigoid implants and 8 were zygomatic implants. The mean age in this group was 55 ± 10.5 years, and 75% of the patients were women. Patients from the control groups received 182 implants, all of them well-centered in the alveolar crest. The mean age in this group was 55.9 ± 7.9 years and 48% of the patients were women Fifty were placed in molar regions and were thus excluded. The patient sample was homogeneous regarding age and sex.

In the test group all the patients received metal-resin screwed prostheses, while in the control group 40% of the prostheses were metal-ceramic and 60% were metal-resin.

**Figure 1:**
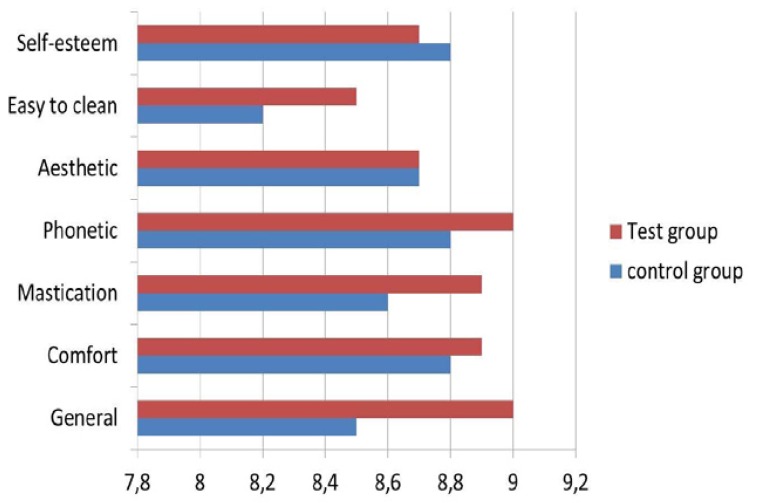
Patient satisfaction.

Mean global and specific satisfaction – except for self-esteem - were superior for the test group than the control group, although differences were not statistically significant. Descriptive and comparative analyses for patient satisfaction are detailed in  Table 1 and figure 1.

**Table 1:**
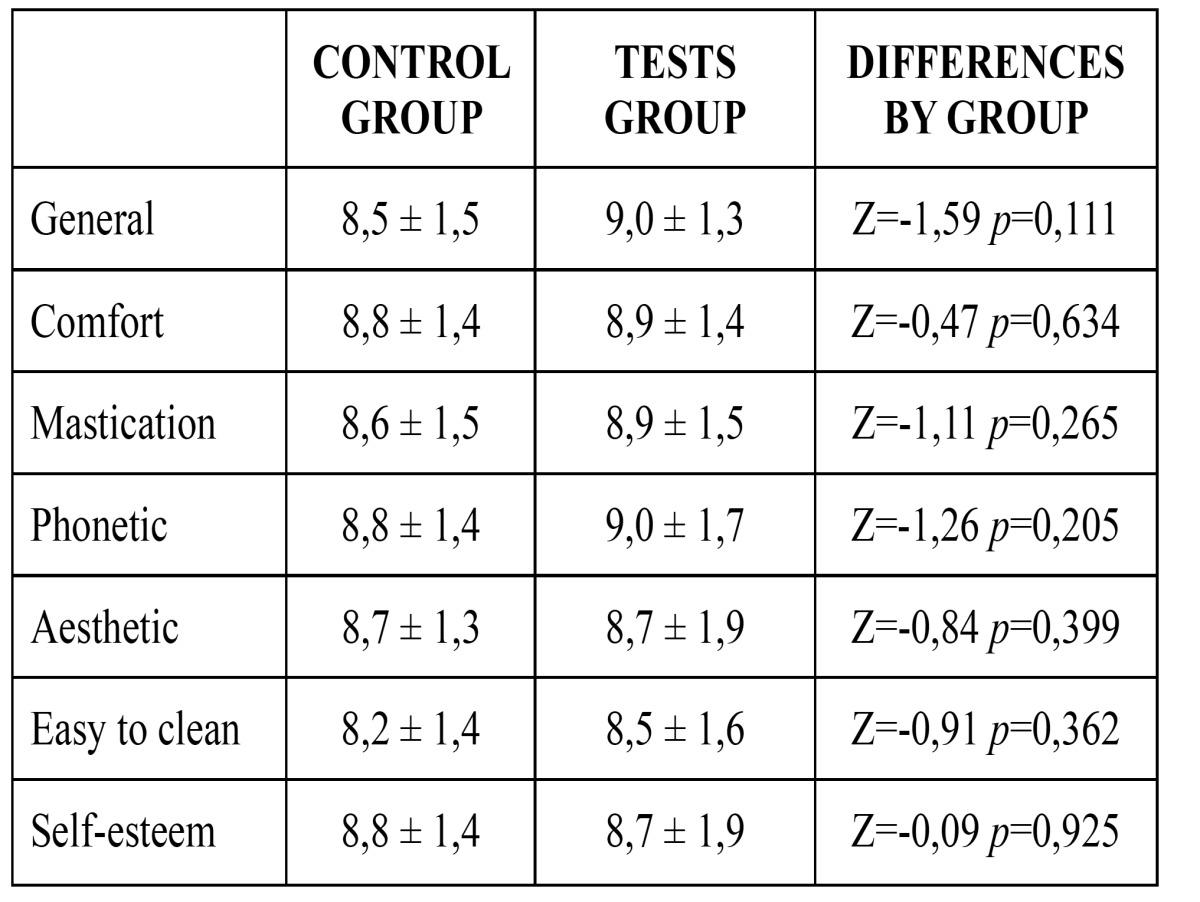
Patient satisfaction.

Regarding quality of life, the reported incidence of problems was lower in the test group for all the studied ítems except for ‘problems at work’. However, differences were not statistically significant in any case. The descriptive and comparative analyses for quality of life are detailed in  Table 2.

**Table 2:**
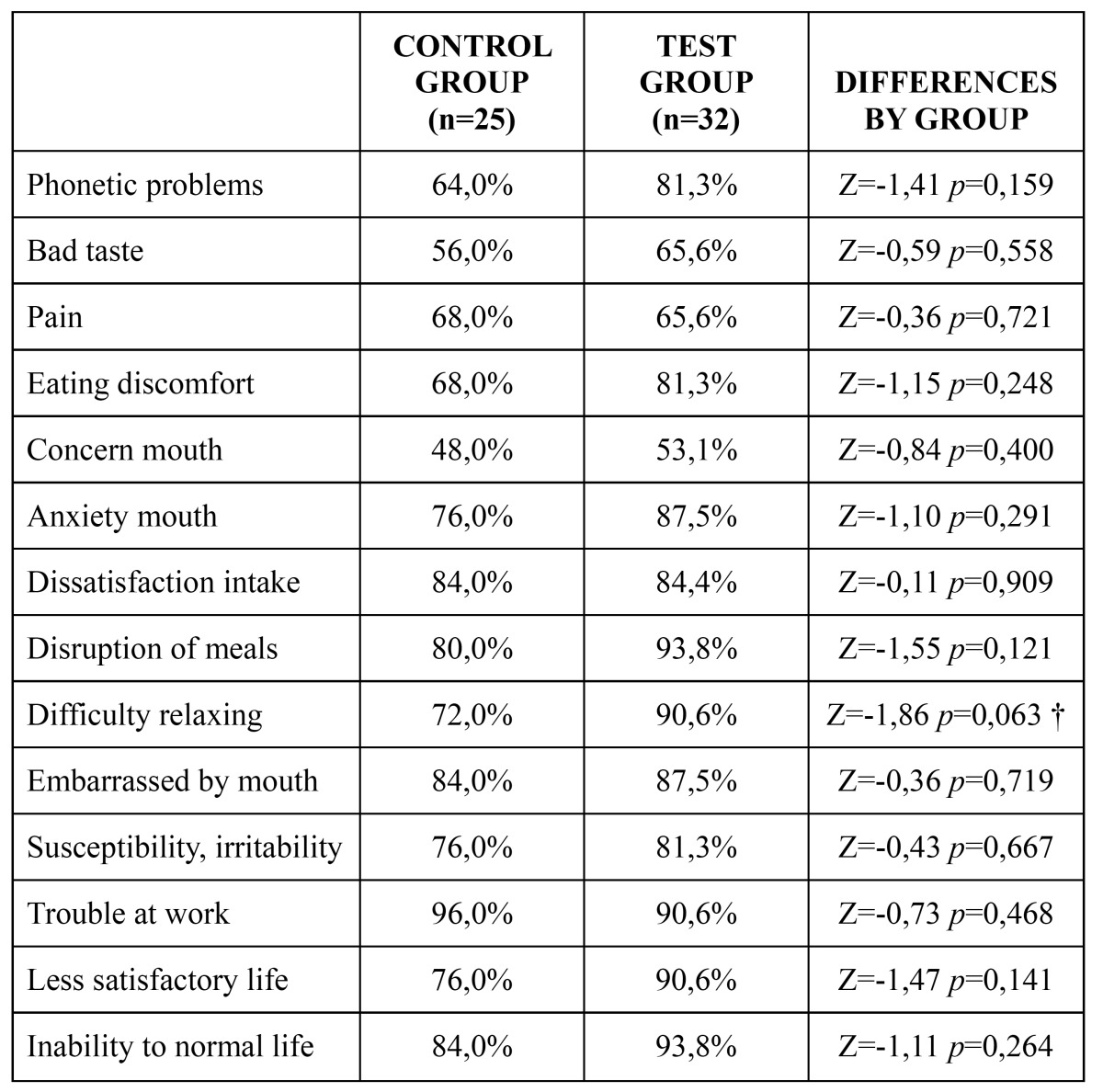
Quality of life.

## Discussion

Present techniques allow us to rehabilitate oral functions and improve the quality of life of edentulous patients, even in presence of severe bone atrophy (14,15). In contrast with bone grafting techniques, placing implants in a palatal position is a surgically less agressive alternative to treat atrophic maxillae (8). However, this technique is associated with a non-ideal prosthetic emergence of the implants. Thus, the suprastructure occupies more palatal space than prostheses supported by conventional well-centered implants, and this may negatively influence patient satisfaction or quality of life. The aim of the present study was to compare patient satisfaction and quality of life with full-arch fixed prostheses supported by palatal positioned implants or by conventional implants placed well-centered in the alveolar crest.

According to the outcomes of the present study, satisfaction and quality of life are very high and very similar in both groups. The statistical analysis yielded no significant differences for any of the assessed items. In fact, for several of the assessed items results were more favorable in the palatal positioned group, although differences were not significant in any case. Peñarrocha *et al*. (16) evaluated the satisfaction of patients with maxillary fixed prostheses supported by conventional and/or zygomatic implants. Forty-six patients participated in the study (23 in each group). The mean level of satisfaction was high; the groups differed significantly only in satisfaction with esthetics. Patients in the zygomatic group had a higher average score for esthetics than those in the nonzygomatic group.The authors conclude that patient satisfaction with zygomatic implant-supported fixed prostheses was similar to that for fixed prostheses supported by conventional implants.

Zembic *et al*. (17) treated 21 edentulous patients with maxillary over dentures that covered or not the palate. Each patient received both types of prostheses during 2 months. No significant differences were found in quality of life between the 2 prostheses, and only for aesthetics and taste was satisfaction better with prostheses that did not cover the palate.

Heydecke *et al*. (18) analyzed 13 patients: 5 patients with over dentures and 8 with fixed prostheses during two months. After two months, patients who took fixed prostheses were placed over dentures and patients wearing over denture were placed fixed prosthesis for another two months. Over dentures had more elevated scores on overall satisfaction, in ability to speak and cleaning ability. Of the 13 patients, 9 preferred to stay with the over denture.

However, Preciado *et al*. (19) conducted a study on 131 patients as compared the quality of life of patients treated with metal-resin hybrid screwed fixed full-arch prosthesis, ceramometalic screwed fixed full-arch prosthesis or ceramometalic screwed fixed partial prosthesis. They observed that the hybrid prosthesis (occupy more palatal space), had worse aesthetic values, form and functional limitation.

## Conclusions

Despite the limitations of the study (retrospective and nonrandomized design) the results suggest that the prosthesis design needed to rehabilitate palatally positioned implants (more coverage of palate) does not lead to lower satisfaction and quality of life of patients, compared to patients treated with implants placed centered and conventional design prostheses that do not cover the palate.
